# Exercise Snacking to Improve Muscle Function in Healthy Older Adults: A Pilot Study

**DOI:** 10.1155/2019/7516939

**Published:** 2019-10-03

**Authors:** Oliver J. Perkin, Polly M. McGuigan, Keith A. Stokes

**Affiliations:** ^1^Department for Health, University of Bath, Bath BA2 7AY, UK; ^2^Centre for Sport, Exercise and Osteoarthritis Research Versus Arthritis, Chesterfield, UK

## Abstract

Loss of muscle mass and strength are seemingly accepted as part of the ageing process, despite ultimately leading to the loss of independence. Resistance exercise is considered to be primary defence against loss of muscle function in older age, but it typically requires access to exercise equipment often in a gym environment. This pilot study aimed at examining the effect of a 28-day, unsupervised home-based exercise intervention on indices of leg strength and muscle size in healthy older adults. Twenty participants were randomly assigned to either maintain their habitual physical activity levels (Control; *n*=10; age, 74 (5) years; body mass, 26.3 (3.5) kg/m^2^) or undertake “exercise snacks” twice daily (ES; *n*=10; age, 70 (4) years; body mass, 25.0 (3.4) kg/m^2^). Both groups consumed 150 g of yogurt at their breakfast meal for the duration of the intervention. Sixty-second sit-to-stand score improved by 31% in ES, with no change in Control (*p* < 0.01). Large effect sizes were observed for the difference in change scores between the groups for interpolated maximum leg pressing power (6% increase in ES) and thigh muscle cross-sectional area (2% increase in ES). The present pilot data suggest that exercise snacking might be a promising strategy to improve leg muscle function and size in older adults and that further investigation into zero-cost exercise strategies that allow high frequency of training is warranted.

## 1. Introduction

Frailty is underpinned by a progressive loss of muscle mass and strength, particularly from the lower limbs, and is associated with increased risk of falls and reduced quality of life [1, 2]. There is a minimum threshold of strength required to complete tasks of daily living independently, and finding means to delay individuals reaching this “frailty threshold” has been identified as an urgent health care priority [3]. With muscle mass lost at 0.5–1% per year after 50 years of age [4] and strength lost even more rapidly [5], modest improvements of a few percent in muscle size and strength from a training programme may, in essence, represent postponement of frailty measurable in years. Crucially, intervention is needed before older adults' functional capacity declines past the point that exercise is no longer a safe means to maintain muscle strength.

Progressive resistance exercise training improves muscle strength in older adults, and it is accompanied by multifaceted improvements in health and function [6, 7]. Traditionally, heavy load resistance training has been regarded as the most effective strategy to increase muscle strength, due to associated neural and hypertrophic adaptations [8, 9]. Recent evidence suggests that low-load resistance training can also be efficacious in increasing muscle strength, particularly in an untrained population, albeit to a lesser degree in comparison to high-load resistance training [10, 11]. Training with low loads and low overall session volume may allow for increased training frequency, as recovery times may be shorter between sessions [12]. Dankel et al. [13] suggests that manipulation of training frequency to maintain overall training volume with more sessions of lower load across a week may even increase hypertrophic training responses. Although this has not yet been borne out empirically [8], it is intuitively appealing to reason that a reduced training session load with short recovery times may suit an older population previously doing no formal exercise because it may overcome some of the barriers to starting an exercise programme [14].

Short bouts of exercise spread across the day, termed “exercise snacks,” have received attention as a time-efficient exercise strategy. Francois et al. [15] identified that exercise snacking before each meal, consisting of six discrete minutes of exercise separated by one minute of rest, improved glycaemic control the following day in middle-aged adults with impaired glucose handling. Jenkins et al. [16] reported improvements in cardiorespiratory fitness in healthy inactive adults performing three sets of maximum effort 60-step stair climbs a day, three times a week, for six weeks. The improvement in exercise tolerance included an increase in maximum power output during a VO_2_ peak test on a cycle ergometer [16]. This suggests that an exercise snacking model may have the potential to improve function beyond just cardiovascular fitness. As such, exercise snacking was examined in the present pilot study with the aim of providing a stimulus to improve leg strength in older adults that could be undertaken in the home on a daily basis without the need for supervision.

The primary aim of the present pilot study was to investigate the effects of four weeks of twice daily “exercise snacking” on maximum number of sit-to-stands from a chair performed in one minute, compared with a control group maintaining their habitual physical activity patterns. The secondary aims were to assess adherence to the exercise snacking intervention, along with the effects on force, velocity, and power, during leg press dynamometry and on whole-body and lower limb anthropometry. The proposed intervention was specifically designed to be suitable to perform in the home environment, without the need for supervision or specific exercise equipment. The overall objective of this pilot study was to inform future work on exercise strategies extending to populations with lifestyle-compromising age-related loss of muscle strength or mass.

## 2. Materials and Methods

**Figure 1:**
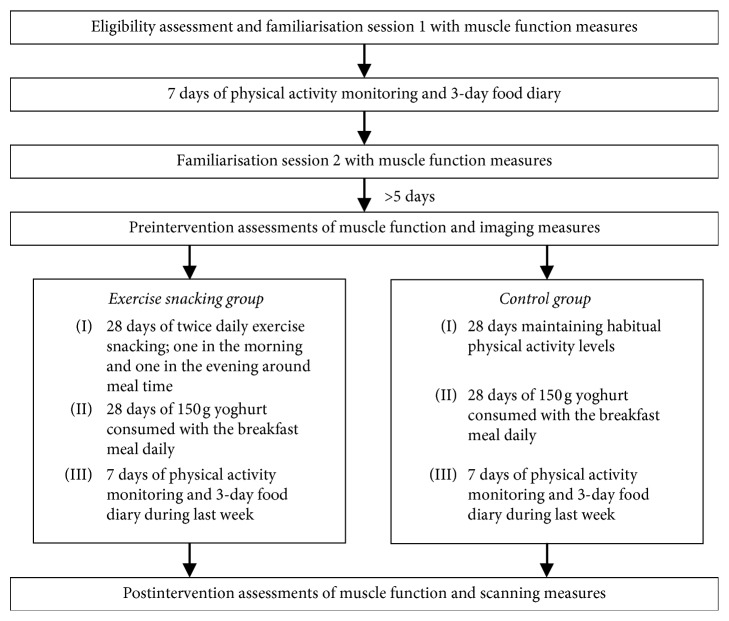
A schematic overview of the pilot study timeline.

A two-group experimental research design was used in this pilot study to examine the effects of twice daily “exercise snacking” on muscle function and size in healthy, community-dwelling older adults. For 28 days, one group undertook a home-based exercise snacking intervention (ES) and the other group acted as a nonexercising control by maintaining habitual physical activity levels. As a control variable, both groups were provided with 150 g of yogurt to consume as part of their breakfast meal for the 28 days, to both act as a “positive control” to reduce participant dropout from the Control group and to achieve optimal dietary protein intake [17]. All participants completed two familiarisation sessions to functional measures used in the pre- and postintervention assessment, separated by at least seven days, with the second session completed at least five days before the preintervention assessments. Function testing and imaging measures were completed on the day before and the day after the 28-day intervention. Between familiarisation sessions and during the last week of the intervention, physical activity and diet were assessed. See Figure 1 for a schematic overview of the pilot study timeline.

### 2.1. Participants

Twenty healthy, community-dwelling, older men and women (65–80 years), not undertaking regular structured exercise, were recruited for the pilot study through local newspaper advertisement and social media. Individuals who were nonsmokers, had BMI of  ≥20 < 30 kg/m^2^, had no contraindications to exercise or recent history of musculoskeletal injury, and had scored 8 or above with no score of zero on any test of the SPPB [18] at the initial screening were invited to take part in the pilot study. Participants were assigned to groups by way of minimisation to limit differences in mean age, BMI, and 60-second sit-to-stand (STS) score at the screening visit, on account of the small sample size [19, 20]. An individual outside of the study team performed participant group allocation. Participant characteristics recorded during screening are presented in Table 1. All participants provided written informed consent. The protocol was approved by the National Health Service (NHS) South West—Frenchay Research Ethics Committee (Ref: 16/SW/0300) and registered on ClinicalTrials.gov (Identifier: NCT02991989).

## 3. Measures

### 3.1. Imaging

Participants arrived at the laboratory for trial days following a 10-hour overnight fast, having drunk 1 pint of water, and having not undertaken exhaustive exercise in the previous 24 hours. Participants were asked to void before weight was measured with electronic scales (BC543, Tanita, Amsterdam, Netherlands). Whole-body composition, whole-body lean mass, and leg lean mass were estimated using a DXA system (QDR software version 12.4.2, Hologic Discovery W, Bedford, MA) by differentiating the fat, bone, and lean (nonbone nonfat) masses. A spine phantom was used for the quality control scan performed at the start of every trial day before participant testing, as per the manufacturer's guidelines. Participants wore the same light clothes for pre- and postintervention trials and removed all metal items. The investigator positioned the participant to be laying supine on the scanning bed such that body regions could be partitioned upon analysis. Manual placement of boundaries between discrete anatomical regions was conducted for all scans by the same investigator (OJP), before analysis using manufacturer's software.

Lower limb (calf and thigh) muscle cross-sectional area (mCSA) was assessed by peripheral quantitative computed tomography (pQCT; XCT3000, StraTec Medizintechnik GmbH, Pforzheim, Germany). During the preintervention trial, tibia (medial knee joint line to medial malleolus) and femur length (greater trochanter to lateral knee joint line) of the dominant leg were measured using a fabric tape measure whilst participants were standing. Scans were performed with the participant laying supine on a bed with leg placed through the scanning gantry and foot strapped into a footplate. Scout scans were performed at the distal ends of the tibia and femur to locate the end of bones, respectively. Single 2D slice scans were performed at 66% of the tibia length proximally from the medial malleolus, and 25% of the femur length proximally to the lateral femoral epicondyle, based on the bone lengths previously recorded. Scan images were analysed using the BoneJ plugin (Version 1.4.2) for ImageJ (1.44p, Wayne Rasband, National Institutes of Health, USA) [21, 22]. Following scanning measures, participants were provided with a breakfast of their choice, which was matched on the postintervention trial day.

### 3.2. Functional Measures

A maximum number of repeated STS in 60 seconds were performed from a chair with a seat height of 44 cm, with arms folded across the chest and reaching full hip and knee extension on standing. During familiarisation, a researcher counted the number of repetitions aloud, with a timing clock in view of the participant. On trial days, participants were not in view of a clock and repetitions were not counted aloud, with participants instructed to complete repetitions at the fastest rate they could manage until told to stop. Immediately on completion of the STS, a rating of perceived exertion (RPE) was assessed using Borg's RPE 15-grade scale [23].

Maximum leg pressing velocity, force, and power characteristics were measured on a seated pneumatic leg press dynamometer (A420, Keiser®, Fresno, CA). Data collection, processing, and analysis were performed as described previously [24]. Briefly, during familiarisation sessions, participants completed tests of one-repetition maximum (1-RM) leg pressing force against self-selected increments in resistance. No emphasis was placed on contraction velocity for the 1-RM test, and participants were instructed to reach 1-RM within 15 repetitions with self-selected interrepetition rest. Participants then performed a series of approximately 10 discrete repetitions, each performed at maximum concentric contraction velocity against an incrementally increasing pedal resistance, up to a resistance equalling the previously achieved 1-RM. On trial days, a set warm-up was performed based on the 1-RM achieved in the second familiarisation, consisting of 5 × 30%-1RM, 5 × 50%-1RM, 2 × 70%-1RM, and 1 × 80%-1RM, followed by five minutes of seated rest. The aforementioned incremental test was then performed, with the tenth repetition at a resistance equal to the 1-RM achieved in the second familiarisation. To extrapolate theoretical maximum contraction velocity (*V*_max_) and force (*F*_max_), linear regression of force and velocity at which peak power of each repetition occurred was calculated. Interpolated peak power (*P*_max_) was determined by numerical differentiation of the second-order polynomial calculated from the force-power profile, i.e., from peak power and the force at the instant of peak power for each repetition [25].

### 3.3. Physical Activity

Free-living physical activity was assessed on seven consecutive days by continuous wear of an armband mounted physical activity monitor (SenseWear, BodyMedia, Inc., Pittsburg, PA, USA). This was undertaken between the familiarisation sessions before the intervention period as a baseline measure and during the last week of the intervention period. Physical activity level (PAL) was calculated as estimated mean daily energy expenditure/resting metabolic rate (estimated using the World Health Organisation equation). Only days with >95% wear time achieved were included in the analysis. Participants were instructed to remove the armband for water-based activity, such as bathing or showering, and any water-based activities were recorded in the logbook.

### 3.4. Diet Records

Three-day weighed diet records (two weekdays and one weekend) were completed by participants. Again, this was undertaken between familiarisation sessions and during the last week of the intervention period. Commercially available online software (v4.312 Nutritics Education, Dublin, Ireland) was used for diet record analysis, all of which was performed by the same researcher (OJP). Mean daily intake of total kcal, carbohydrate (CHO), protein (PRO), and fat were obtained and calculated relative to body mass using screening body mass and postintervention body mass for baseline and intervention dietary records, respectively. Between pre- and postintervention trials, participants consumed 150 g of yogurt (Natural flavour, Skyr, Arla®; 98 kcal, 0.3 g fat, 6 g carbohydrate, 16.5 g protein) as part of their breakfast meal. Participants were provided with food weighing scales and a logbook to record whether the full 150 g of yogurt had been consumed each day and deliberately not given any further instruction concerning dietary intake.

### 3.5. Intervention

The Control group was asked to continue with their habitual physical activities for 28 days. The exercise snacking group was asked to perform two bouts of “exercise snacking” per day, once in the morning and once in the evening. Exercise snacking bouts consisted of five exercises, each undertaken for one minute with the aim to complete as many repetitions as possible in that minute. Between each exercise, participants rested for one minute. The exercises were STS from a chair, seated knee extensions of alternating legs, standing knee bends of alternating legs, marching on the spot, and standing calf raises (see Supplemental Figures [Supplementary-material supplementary-material-1]–[Supplementary-material supplementary-material-1], respectively). Participants were advised to hold onto a chair for stability during standing exercise if they felt the need to. The STS exercise was performed first, with the number of repetitions completed recorded in a provided logbook as a means to assess adherence. Any adverse events during the intervention period for either group were to be recorded in a logbook, regardless of whether they were related to the intervention.

### 3.6. Statistical Analysis

Shapiro–Wilks tests of normality were performed on participant characteristic data recorded at screening (age, body mass, BMI, and STS score) due to the small sample size. Participant characteristic variables were normally distributed, thus compared with independent-samples *t*-test. Outcome variables were analysed with a two-way repeated measures ANOVA, and where there was a significant interaction or time effect was observed, a Holm–Bonferroni *post hoc* test performed. Statistical significance was accepted at *p* < 0.05. To infer the magnitude of differences between the groups, Hedges *g* effect size for difference in change scores between the groups was calculated, to account for low sample size [26]. Effect sizes were classed as small (0.2), moderate (0.5), and large (0.8) according to Cohen [27]. Data are presented as mean (standard deviation); ANOVA and *post hoc* analysis were performed using SPSS v22.0 (SPSS Inc., Chicago, IL), and effect size analysis was performed using Microsoft® Excel® 2016.

## 4. Results

### 4.1. Functional Measures

**Figure 2:**
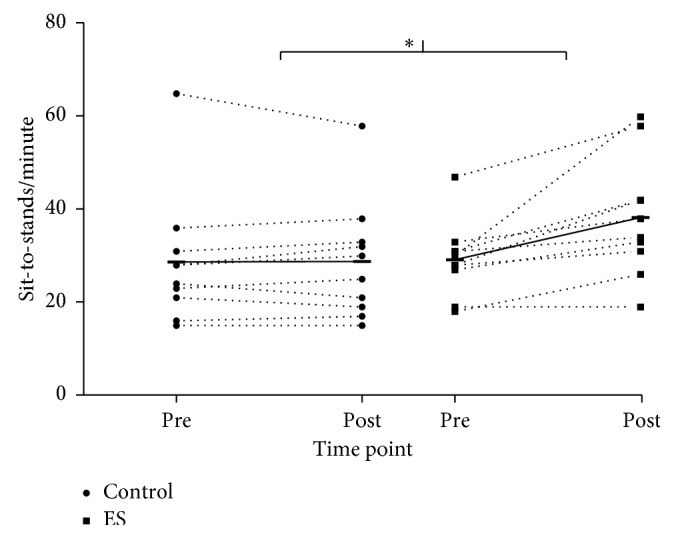
Individual changes in sit-to-stand score from pre- to postintervention of either 28 days of yogurt at breakfast only (Control) or yogurt at breakfast and exercise snacking twice daily (ES). Horizontal bars connected with solid lines display group mean. ^*∗*^denotes significant difference in change score between the groups (*p* < 0.01).

Adherence to the ES intervention was 98% (2 (1) sessions out of 56 sessions missed), and no adverse events occurred during the intervention period in either group. Pre- to postintervention STS scores were significantly increased (*p* < 0.01) in the ES group (29 (8) to 38 (13)), compared with the Control group (29 (14) to 29 (13)). There was a large between-group effect size of *g* = 1.40 for the difference in STS change scores (Figure 2). There was no significant change in RPE for the STS pre- and postintervention in either group (Control: 13 (3) to 14 (2); ES: 13 (2) to 14 (2)). There were no significant time or interaction effects for *V*_max_, *F*_max_, or *P*_max_ (Table 2). Effect sizes for change scores between the groups were moderate for *V*_max_ and *F*_max_ (*g* = 0.62 and *g* = 0.49, respectively) and large for *P*_max_ (*g* = 0.81).

### 4.2. Anthropometry

One participant from the Control group was removed from DXA analysis due to movement artefact on one scan. As shown in Table 3, there were no significant changes in body mass, or DXA measured % body fat, total lean mass, or lean leg mass in either group, but a moderate effect size for the difference in leg lean mass change scores (*g* = 0.68).

**Figure 3:**
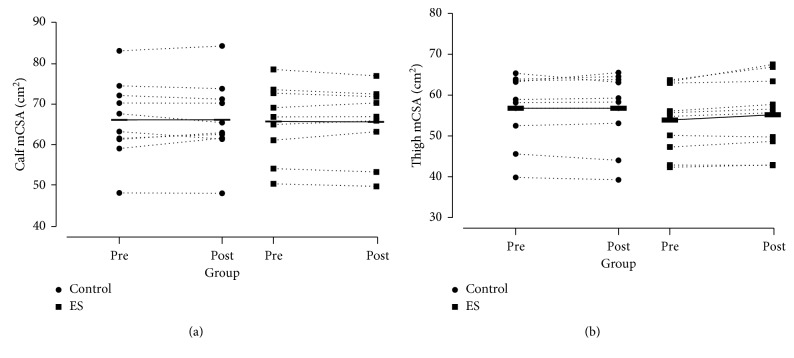
Individual changes in pQCT measured (a) calf muscle at 66% tibia and (b) thigh muscle group at 25% femur, pre- and postintervention of either 28 days of yogurt at breakfast only (Control) or yogurt at breakfast and exercise snacking twice daily (ES). Horizontal bars connected with solid lines display group mean.

One participant from the ES group was removed from the calf pQCT scan analysis, and one participant from the Control group was removed from the thigh pQCT scan analysis, both due to movement artefact. Calf mCSA did not change significantly for either group, with an effect size for difference in the change scores between the groups being *g* = 0.10. There were no statistically significant changes in thigh mCSA in either group, although there was a large effect size for the difference in change scores between the groups (*g* = 0.96) with an increase of 2% in the ES group (see Figure 3).

### 4.3. Physical Activity and Diet

There were no changes in PAL in either group from baseline assessment to the last week of the intervention, nor were there changes in total energy (kcal/kg/day) or carbohydrate intake (g/kg/day). There were significant time effects for an increase in daily protein intake (*p* < 0.01) and decrease in daily fat intake (*p* < 0.05). Because there were no differences between the groups, the effect size for the pooled change score (pre- to postintervention for all participants) were moderate for dietary protein and fat intake (*g* = 0.71 and *g* = 0.60, respectively) (see Table 4).

## 5. Discussion and Implications

The impact of undertaking 28 days of twice daily home-based exercise snacking, supplemented with 150 g of yogurt at breakfast, on lower limb muscle function and anthropometry was explored in healthy older adults. Adherence to the exercise regime was very high (98%), and participants in the ES group showed marked improvements in the number of sit-to-stands performed in 60 seconds, with no improvement in the Control group. Large effect sizes were also observed in the change scores for interpolated maximum leg pressing power and thigh muscle cross-sectional area, albeit the absolute increases in these variable appear modest.

The exercise snacking regime consisted of five leg exercises; each completed twice a day across two bouts, with the aim to complete as many repetitions of each exercise as possible in a minute with no external load above body weight. This mode of exercise deviates from successful home-based exercise programmes explored previously; primarily, in that, all exercise was nonloaded, participants undertook exercise twice a day, there were no supervised exercise sessions in the home, and the programme lasted only four weeks [28]. The ES group showed significantly improved STS score, with the 31% improvement in the STS in 60 seconds in the present pilot study being remarkably similar to the 30% improvement in 30-second STS score observed after six weeks of resistance training in older adults by Cavani et al. [29]. With 60-second STS being one of the exercises completed twice daily for the ES group, this was not unexpected, even though the conditions between exercise bouts and testing sessions were deliberately different (self-timed vs. no information of time remaining in the test). Although the improvement in STS is likely largely due to task-specific training, the movement pattern is nonetheless relevant for tasks of daily living [30]. Given that the mode of training applied no external load beyond body weight and did not require participants to exercise to momentary failure, improvements in leg press *V*_max_ and *F*_max_ of 3% and 5%, respectively, along with a trend toward significant increases in *P*_max_ (6% increase; *p*=0.09) were perhaps not expected and provide an indication of the potential efficacy of this type of “little and often” intervention.

As a point of comparison, in the study by Bean et al. [31], older adults trained three times a week for 12 weeks, completing three sets of 10 repetitions of maximum concentric contraction speed exercises similar to those of the present pilot study, whilst wearing a weighted vest. The weighted vest group increased maximum leg press power by 12%, also assessed with pneumatic leg press dynamometry. Although the present pilot study employed an intervention only one-third of the programme duration as the aforementioned study, and without external loading, a 6% increase in *P*_max_ was observed. Whether functional improvements of the exercise snacking regime over longer training durations would continue to increase with the only element of progression being completion of more repetitions in a minute cannot be known. It should be noted that although the participants in the present pilot study were previously undertaking no regular structured exercise, they were healthy and not functionally impaired, so were perhaps more physiologically receptive to the training stimulus provided than a frail or clinical population might be [32]. Nonetheless, increases in *F*_max_ and *P*_max_ of 5% and 6%, respectively, represent changes with real-world relevance given the estimated annual loss of muscle strength of 1–5% described by Seene and Kaasik [33], particularly as the strength and power gains were achieved in four weeks with a zero-cost exercise intervention. However, whether these task-specific increases in strength and power lead to clinically relevant improvements in outcomes, such as delaying dynapenia/sarcopenia or frailty, would require further investigation and long-term follow-up.

There were some small but noteworthy changes in anthropometric measures of the legs following the ES intervention. In particular, leg lean mass measured by DXA increased by 1% (*g* = 0.68) and thigh mCSA increased by 2% with a large effect size (*g* = 0.98). In comparison to an effect size of 0.39 (0.17) (95% CI: 0.05, 0.73) for hypertrophy induced by low-load resistance exercise training calculated in a recent meta-analysis by Schoenfeld et al. [11], the potential increase in muscle size observed in present pilot is notable. No mechanisms for an increase in muscle size were examined in this work, but it could be in part due to the comparatively high frequency of training, although this of course can only be speculated due to the scarcity of studies using twice daily exercise programmes [8, 13]. It is also possible that any hypertrophy observed in the present pilot study was in part due to the additional protein ingested at breakfast via the yogurt supplement. This notion would be supported by the evidence of Mamerow et al. [34], albeit in a younger population, that 24-hour muscle protein synthesis rate was greater with an even distribution of protein throughout the day compared with a more traditional, evening heavy, protein distribution. Although it is encouraging that two independent measurement techniques concomitantly suggest that a short-term exercise snacking intervention may have potential to increase leg muscle size, the absolute changes were small, and exercise-induced oedema from the previous day's exercise cannot be ruled out as a potential confounder in this instance [35]. However, this seems unlikely given the nature of the exercise snacking bouts, the fact that participants would have been accustomed to the exercise after 28 days, and that the increase in mCSA was not observed in the calf muscle group that was also measured by pQCT [36].

Both groups modestly increased daily protein intake per kilogram of body mass by 17% and 19% during the intervention (Control and ES, respectively), going from 1.05 (0.20) g/kg/day to 1.24 (0.32) g/kg/day (pooled mean). Although the older adults in the present pilot study were previously consuming over-the-recommended daily allowance (RDA) of protein, Phillips et al. [17] present convincing rationale that the RDA may not represent an optimal daily protein intake for older adults in particular. The suggested range of 1.2 to 1.6 g/kg/day as a more appropriate daily protein intake was achieved in the present pilot study with the addition of 16.5 g of dairy protein at breakfast. However, further inspection of the absolute change in dietary protein consumption highlighted that total protein intake increased by ≈10 g/day. There was not an increase in daily protein intake equal to the amount contained in the yogurt possibly because protein that would have been included in the breakfast meal may have been replaced with the yogurt. This is potentially important on a per meal basis, as larger protein doses are required to maximally stimulate muscle protein synthesis in older adults. Moreover, this potentially serves to highlight the challenges with supplementing older adult diets with additional protein. In any case, whether an extra 10 g/d protein intake would lead to clinically relevant outcomes in an older population is questionable. The lack of increase in strength or hypertrophy in the Control group support the work of Kim et al. [37], finding that even adjusting protein distribution for 8 weeks without the addition of exercise does not increase muscle mass or strength in older adults. Importantly, this was despite achieving over 1.2 g/kg/day of protein, highlighting the importance of a combination of exercise and nutrition to address muscle loss with ageing However, the duration of the intervention in the present pilot study was very short, and the use of only three-day diet records are often criticised for a bias toward under reporting [38]. Consequently, these data should not be seen to undermine the potential health and body composition benefits of a longer-term increase in dietary protein intake for older adults not meeting recommended quantities of dietary protein intake [39].

There are a number of crucial considerations when contextualising the present findings. Most obviously, the pilot study design employed cannot support the efficacy of exercise snacking without dietary protein supplementation. The addition of two further groups (a true nonexercising control group and an exercise snacking without yogurt group) would shed light on the importance of the additional protein at breakfast, but it would require a large increase in sample size. Moreover, based on the effect size of the present pilot study data, power calculations (G*∗*power, Version 3.1.9.4) indicated that for statistically significant differences in *P*_max_ (*α*=0.05 and power = 0.8), 27 participants per group would have been required using the current research design. Equally, group sizes of 19 would have been required for statistically significant differences in thigh mCSA. By increasing sample size, a traditional randomisation strategy to allocate participants to study groups could have been appropriate, whereas due to a small sample size, minimisation was implemented as means to reduce chance difference in baseline characteristics of groups in this present pilot study [20]. Although the adherence to the exercise programme was very high, it cannot be assumed that this would persist longer than four weeks or in other older populations [40]. Investigation of physiological mechanisms to support the strength and hypertrophic gains observed in the present findings may also allow for further optimisation of the exercise regime itself, creating a potentially more efficacious training stimulus. In the same vein, a longer-term follow-up would also be required to establish whether the physiological adaptions might continue to occur if the exercise stimulus were to be maintained. It should of course not be overlooked that high-load resistance training has superior effects on increasing muscle mass and strength [11, 41], potentially through more pronounced neural adaptations [42], and more easily accommodates progressive overload to facilitate continuous improvements in muscle mass and strength. However, accepting that access and cost of exercise participation and lack of knowledge of exercise modalities are often key barriers to exercise in older adults [43], very simple home-based exercise snacking-style regimes are a promising strategy to engage older adults in exercise.

## 6. Conclusions

In conclusion, although underpowered to show statistically significant changes, the present study highlights the potential efficacy of a 28-day, home-based “little and often” exercise snacking programme, for improving leg power and muscle size in healthy older adults. Along with marked task-specific improvements in sit-to-stand score, indications that maximum leg press force and power may also improve modestly with exercise snacking demonstrate transferability of the training. Whether these changes represent potentially clinically relevant improvements in function requires further investigation. Although more research is certainly needed to explain the mechanisms by which such a short-term exercise snacking regime may improve muscle strength and size, understanding the real-world acceptability of this exercise strategy could help provide easy-to-act-upon recommendations for older adults to maintain function into later life.

## Figures and Tables

**Table 1:** Participant characteristics at screening.

	Control (*n* = 10; ♀ = 7)	ES (*n* = 10; ♀ = 7)
Age (years)	74 (5)	70 (4)
Body mass (kg)	70.9 (11.9)	69.7 (9.9)
BMI (kg/m^2^)	26.3 (3.5)	25.0 (3.4)
SPPB score	11 (1)	12 (1)
STS score at screening	29 (12)	29 (10)
PAL at screening	1.63 (0.19)	1.70 (0.14)

Data are presented as mean (standard deviation). ES: exercise-snacking group; BMI: body mass index; SPPB: short physical performance battery; STS: 60-second sit-to-stand test; PAL: physical activity level (ratio of total energy expenditure to basal metabolic rate).

**Table 2:** Summary data of leg pressing outcome measures.

	Group	Pre	Post	%Δ	*p*	*g*
*V*_max_ (m/s)	Control	1.61 (0.29)	1.56 (0.24)	−3	0.19	0.62
	ES	1.75 (0.34)	1.81 (0.23)	3		
*F*_max_ (N)	Control	950 (290)	929 (170)	−2	0.29	0.49
	ES	984 (249)	1032 (289)	5		
*P*_max_ (W)	Control	370 (98)	363 (86)	−2	0.09	0.81
	ES	446 (170)	472 (166)	6		

ES: exercise snacking group; *V*_max_: extrapolated maximum leg pressing velocity; *F*_max_: extrapolated maximum leg pressing force; *P*_max_: interpolated maximum leg pressing power. Data are presented as mean (SD); %Δ, pre- to postchange within groups, *p* values for interaction effect from two-way repeated measures ANOVA, and Hedges *g* effect size of difference in changed scores between the groups.

**Table 3:** Summary of body mass and dual energy X-ray absorptiometry measures.

	Group	Pre	Post	%Δ	*p*	*g*
Body mass (kg)	Control	70.5 (11.2)	70.3 (11.4)	0	0.64	0.27
	ES	69.0 (10.0)	69.0 (10.0)	0		
% body fat	Control (*n*=9)	35.1 (7.4)	35.2 (6.8)	0	0.34	0.48
	ES	34.0 (7.0)	33.7 (7.0)	−1		
Lean mass (kg)	Control (*n*=9)	44.8 (8.6)	44.6 (8.4)	0	0.37	0.44
	ES	44.9 (6.5)	45.0 (6.4)	0		
Leg lean mass (kg)	Control (*n*=9)	15.3 (2.4)	15.3 (2.3)	0	0.17	0.68
	ES	15.3 (2.0)	15.5 (2.2)	1		

ES: exercise snacking group. Data are presented as mean (SD); %Δ, pre- to postchange within groups; *p* values for interaction effect from two-way repeated measures ANOVA, and Hedges *g* effect size of difference in changed scores between the groups. Group size was *n*=10 unless stated otherwise.

**Table 4:** Summary data of dietary intake preintervention and during the intervention.

	Group	Pre	During	%Δ	*p*	*g*
Energy intake (kcal/kg/day)	Control	28 (7)	26 (6)	−9	0.73	0.18
	ES	28 (6)	27 (9)	−4		
CHO intake (g/kg/day)	Control	3.02 (0.88)	2.82 (0.84)	−7	0.84	0.09
	ES	2.95 (1.21)	2.66 (0.85)	−10		
PRO intake (g/kg/day)	Control	1.01 (0.19)	1.17 (0.30)	17	0.73	0.16
	ES	1.10 (0.21)	1.30 (0.34)	19		
Fat intake (g/kg/day)	Control	1.11 (0.47)	0.83 (0.28)	−25	0.67	0.19
	ES	1.32 (0.40)	1.11 (0.40)	−15		

ES: exercise snacking group; CHO: carbohydrates; PRO: protein. Data are presented as mean (SD); %Δ, pre- to postchange within groups; *p* values for interaction effect from two-way repeated measures ANOVA, and Hedges *g* effect size of difference in changed scores between the groups.

## Data Availability

All data supporting this study are openly available from the University of Bath data archive at https://doi.org/10.15125/BATH-00701

## Supplementary Materials

Supplementary Materials Supplementary Figure S1: sit-to-stand (STS) performed from a chair with arms folded across the chest, reaching full hip extension at the top of the stand. Supplementary Figure S2: seated knee extensions, with repetitions performed unilaterally alternating the moving leg, aiming to fully extend the knee at the end of the movement. Supplementary Figure S3: standing knee bends, with repetitions performed unilaterally alternating the moving leg, aiming the shank to reach parallel with the floor whilst the thigh stays vertical. Supplementary Figure S4: marching on the spot, with hands held at waist height (or one holding a chair for balance if required) and aiming to bring thighs up to parallel with the floor. Supplementary Figure S5: standing calf raises, performed bilaterally holding onto a chair for balance, aiming to rise as high onto tiptoes as possible and returning heels to the floor between repetitions.
