# Supplementary material for: Exercise Snacking to Improve Muscle Function in Healthy Older Adults: A Pilot Study
Source: J Aging Res. 2019 Oct 3;2019:7516939. doi: 10.1155/2019/7516939 (PMC6794984; doi:10.1155/2019/7516939)
Supplement: Supplementary Materials — Supplementary Figure S1: sit-to-stand (STS) performed from a chair with arms folded across the chest, reaching full hip extension at the top of the stand. Supplementary Figure S2: seated knee extensions, with repetitions performed unilaterally alternating the moving leg, aiming to fully extend the knee at the end of the movement. Supplementary Figure S3: standing knee bends, with repetitions performed unilaterally alternating the moving leg, aiming the shank to reach parallel with the floor whilst the thigh stays vertical. Supplementary Figure S4: marching on the spot, with hands held at waist height (or one holding a chair for balance if required) and aiming to bring thighs up to parallel with the floor. Supplementary Figure S5: standing calf raises, performed bilaterally holding onto a chair for balance, aiming to rise as high onto tiptoes as possible and returning heels to the floor between repetitions. [file 7516939.f1.pdf]

Supplementary Material

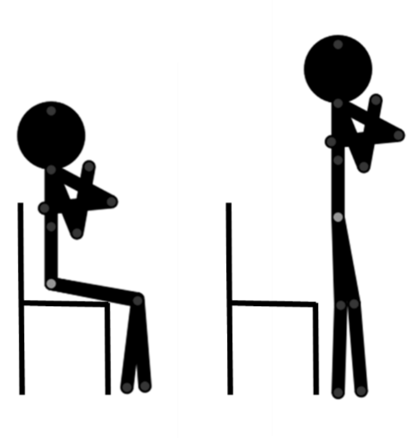

Supplementary Figure S1. Sit-to-stand (STS) performed from a chair with arms folded across the chest, reaching full hip extension at the top of the stand.

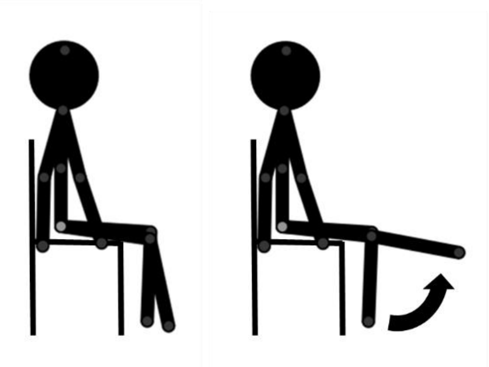

Supplementary Figure S2. Seated knee extensions, with repetitions performed unilaterally alternating the moving leg, aiming to fully extend the knee at the end of the movement

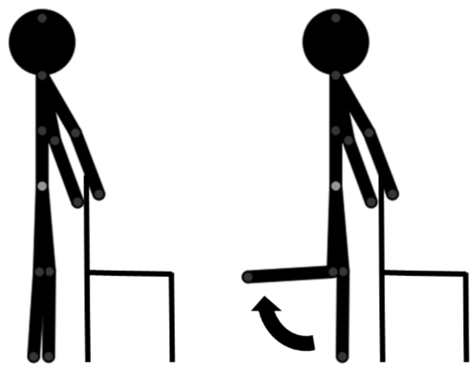

Supplementary Figure S3. Standing knee bends, with repetitions performed unilaterally alternating the moving leg, aiming the shank to reach parallel with the floor whilst the thigh stays vertical.

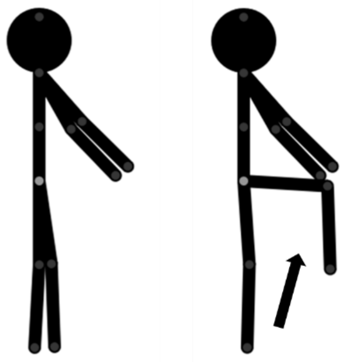

Supplementary Figure S4. Marching on the spot, with hands held at waist height (or one holding a chair for balance if required) and aiming to bring thighs up to parallel with the floor.

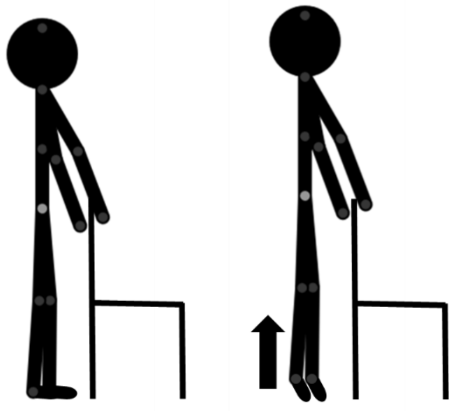

Supplementary Figure S5. Standing calf raises, performed bilaterally holding onto a chair for balance, aiming to rise as high onto tiptoes as possible and returning heels to the floor between repetitions.
